# Attending to child agency in paediatric palliative care consultations: Adults’ use of tag questions directed to the child

**DOI:** 10.1111/1467-9566.13437

**Published:** 2022-01-28

**Authors:** Katie Ekberg, Stuart Ekberg, Lara Weinglass, Anthony Herbert, Johanna Rendle‐Short, Myra Bluebond‐Langner, Patsy Yates, Natalie Bradford, Susan Danby

**Affiliations:** ^1^ School of Early Childhood and Inclusive Education Queensland University of Technology Brisbane City Queensland Australia; ^2^ School of Psychology & Counselling Queensland University of Technology Brisbane City Queensland Australia; ^3^ Centre for Healthcare Transformation Queensland University of Technology Brisbane City Queensland Australia; ^4^ Children’s Health Queensland Hospital and Health Service Brisbane City Queensland Australia; ^5^ Centre for Children's Health Research Brisbane City Queensland Australia; ^6^ School of Nursing Queensland University of Technology Brisbane City Queensland Australia; ^7^ College of Arts and Social Sciences Australian National University Canberra Australian Capital Territory Australia; ^8^ Louis Dundas Centre for Children’s Palliative Care University College London Great Ormond Street Institute of Child Health London UK; ^9^ Department of Sociology, Anthropology and Criminal Justice Rutgers University Camden New Jersey USA; ^10^ Faculty of Health Queensland University of Technology Brisbane City Queensland Australia; ^11^ Australian Research Council Centre of Excellence for the Digital Child Queensland University of Technology Brisbane City Queensland Australia

**Keywords:** child agency, conversation analysis, paediatric palliative care, tag questions

## Abstract

Children's agency in their own lives is increasingly recognised as important, including within paediatric health care. The issue of acknowledging child agency is complex in the context of paediatric palliative care, where children have serious and complex conditions that often impact their ability to verbally communicate with others. This study explores how clinicians and parents/guardians direct talk towards a child patient when they are present in a consultation. Conversation analysis methods were used to examine 74 video‐recorded paediatric palliative care consultations. Detailed turn‐by‐turn examination of the recorded consultations identified the recurrent use of a practice described by linguists as a ‘tag question’, which follows some statement (e.g. ‘he loves that, don't ya’). Both clinicians and parents/guardians often directed these tag questions towards the child patient. Analysis demonstrated how these tag questions: (1) validated the child's epistemic authority over what was being said and (2) made a child's response a possible, but not necessary, next action. The findings are discussed in relation to the sociology of child agency and how this agency is acknowledged and displayed within and through social interaction. This research provides direct evidence of children's competence as informants about their own symptoms.

## INTRODUCTION

Children diagnosed with life‐limiting conditions confront circumstances that are vastly different from adults with life‐limiting conditions (Field & Behrman, 2003; Klick et al., 2014; Levine et al., 2013; Williams‐Reade et al., 2015). Not only can they be diagnosed with different conditions (Baker et al., 2015; Bradford et al., 2014; Hynson et al., 2003), but also they often respond differently to these conditions, and their ongoing physical, emotional, social, cognitive and spiritual development makes their circumstances fundamentally different to adults with life‐limiting conditions (Field & Behrman, 2003; Klick et al., 2014; Rushton & Catlin, 2002; Williams‐Reade et al., 2015). In addition, parents or guardians typically have a social and legal responsibility for a child and their care (Baker et al., 2015; Bluebond‐Langner et al., 2010; Field & Behrman, 2003; Levetown, 2008; Lipstein et al., 2012; Ruhe et al., 2014). Within this complex dynamic, there is a real risk that children's agency and perspectives may be overlooked. This study reports the findings of detailed examination of a communication practice that directly attends to a child's agency within paediatric palliative care consultations involving children with life‐limiting conditions.

The importance of supporting children's agency in their own lives has been given increasing recognition over the past three decades. This perspective is enshrined through the United Nations Convention on the Rights of the Child (UNCRC), which affords children legal rights to participate in practices and decisions that relate to matters that affect their own lives (United Nations, 1989). Viewing children as active social agents require viewing them as competent and capable members of society (Prout & James, 2015). Recognising and understanding the agency of children have become increasingly prominent in academic research. In the 1970s, ethnomethodologists, including Speier (1971), Mackay (1974) and Bluebond‐Lagner (1978), valued and recognised that children were competent to accomplish their own agendas. Since then, in the 1990s, a new sociology of childhood viewed children as active in the construction and determination of their own social lives, rather than passive subjects of social structures and processes (Danby, 2002; Danby & Baker, 2000; Hutchby & Moran‐Ellis, 1998; Moran‐Ellis, 2010; Prout & James, 2015). A key way to explore child agency is through interactional and ethnographic methods (Danby & Baker, 1998; Moran‐Ellis, 2010; Prout & James, 2015; Theobald & Danby, 2020). Agency is produced in and through children's social interactions with other children and with adults. Conversation analysis, in which naturally occurring social interactions are systematically examined in minute detail, has proven useful for exploring how children's agency is co‐constructed through unfolding sequences of talk with children in various settings, including institutional settings such as education (Butler, 2008; Church & Bateman, 2019; Cobb‐Moore et al., 2009; Danby et al., 2016; Houen et al., 2016), children and young people's helplines (Butler et al., 2009) and health care (Clemente, 2009, 2015; Clemente et al., 2008, 2012; Jenkins et al., 2020). The current study contributes to research in this area.

### Child agency in paediatric health care appointments

One institutional setting where children can be involved in conversations about themselves is in health care. Issues of child agency and competency are fundamental in health care, as a patient is, ultimately, the person who has direct subjective experience of their health, wellbeing and the symptoms that are the typical reason for seeking health care (Arminen, 2017; Butler et al., 2015; Emmison & Danby, 2007; Heritage & Raymond, 2005; Iversen, 2019; Jenkins et al., 2020; Muntigl et al., 2014; Sacks, 1984b). Children's talk, however, typically only accounts for around 5% of the conversation during paediatric consultations, with most talk being a dyadic exchange between a health‐care professional and the child's parent or guardian (Cahill, 2010; Cahill & Papageorgiou, 2007). Clinicians and parents or guardians face the challenge of providing opportunities for children to participate but without imposing burdens on the child that may make them unwilling or unable to actively participate in the consultation (Clemente et al., 2008, 2012).

Recent conversation analytic research has identified ways clinicians can encourage child participation in health‐care consultations (Cahill, 2010; Clemente et al., 2008, 2012; Stivers, 2001). Children were found to be more likely to contribute to the history‐taking phase of a consultation when they were explicitly selected as the recipient of a question, through practices such as using the child's name or gazing at them (Cahill, 2010; Stivers, 2001). Clinicians can also promote a child's account of their symptoms by using communication practices such as asking open‐ended questions and waiting for a response, renewing open‐ended solicitations, shifting to closed‐ended questioning, asking for the child's permission to solicit parental assistance, using expressions that display the child as primary ‘knower’ of their symptoms and using nonfocussed questioning (Clemente, 2009; Clemente et al., 2012). These communication practices of using open or nonfocussed questions allow the child the most choice in how they frame their response. Nevertheless, such practices also place the most demands on the child, whether they be cognitive or interactional, which risks the possibility that no response will be produced by a child or that a parent will respond instead of the child (Clemente, 2009; Clemente et al., 2012).

In addition to ways that the conduct of adults can encourage child participation, children themselves instigate talk within paediatric consultations. For instance, children display their rights as the person who has direct subjective experience of their symptoms (Clemente, 2009; Jenkins et al., 2020). Children have been found to instigate the sharing of medically relevant information that lies within domains where they have direct subjective experience (Jenkins, 2015; Jenkins et al., 2020). Children both invite and exclude parental participation to support their symptom descriptions as needed or wanted, and sometimes reject parents or guardians’ formulations of their experience (Clemente, 2009; Jenkins, 2015). This research provides direct evidence of ways children display competence as informants about their own symptoms. Even when they withhold responses, children display their competence in using practices that do not align with the agendas of others (Bluebond‐Langner, 1978; Clemente, 2015; Hutchby, 2002).

The issue of acknowledging child agency and encouraging child involvement in their health‐care encounters becomes even more complex in the context of paediatric palliative care, where many children have serious and complex conditions that influence their ability to verbally communicate with others. Children remain the ultimate authorities on their own experiences but may not always be able to communicate these experiences in the same way as a child without a serious and complex condition. Finding ways to acknowledge and include children within consultations is especially challenging for clinicians and parents or guardians in this setting. There is scant direct empirical evidence of how clinicians and parents or guardians communicate with child patients within paediatric palliative care (Ekberg et al., 2018). In addition, the most recent handbook on communication in palliative care gives no attention to communicating with child patients (Kissane et al., 2017). While this topic was addressed in a book prepared through the US Institute of Medicine (Field & Behrman, 2003), it reports expert opinions on how to manage the challenges of involving child patients, such as the prognosis of their condition. The current study moves beyond this, to directly study how children are involved in paediatric palliative care consultations.

This study addresses the gap in knowledge about children's involvement in paediatric palliative care consultations by examining a frequently observed communication practice used by clinicians and parents/guardians during paediatric palliative care consultations when the child patient was present. This practice was the use of tag questions directed to the child.

### Tag questions in social interaction

Linguists define tag questions as having a combination of an anchor (most often a declarative claim) and a tag (Kimps, 2018; Tottie & Hoffmann, 2006). Tag questions usually involve an anchor with positive polarity followed by an interrogative with negative polarity, such as ‘You are, aren't you’ (Kimps, 2018; Tottie & Hoffmann, 2006). In the current study, invariant tag questions (Quirk et al., 1985) that are appended to an anchor and invite a listener's response are also included for analysis (e.g. ‘right?’, ‘eh?’, ‘hey (name)?’). The majority of tag questions have falling intonation on the tag component (68%) rather than having rising intonation as might be expected for a question (Kimps, 2018).

A key action of the tag component in a tag question is that it orients to the knowledge status of someone other than the current speaker (Heritage & Raymond, 2005; Kimps, 2018). Depending on its polarity, the tag expresses a degree of certainty or uncertainty towards the anchor statement. Importantly, the tag signals epistemic authority, on the basis of knowledge or experience, in relation to the anchor statement. Tag questions can be produced in either initiating or responsive actions. Some tag questions are used in initiating actions when reporting an ‘AB‐event’, for which Person A and Person B share knowledge or experience (Heritage, 2012; Kimps, 2018; Labov, 1972). Other tag questions are produced in initiating actions that relate to ‘B‐events’, for which Speaker B (the recipient) is the ultimate epistemic authority (Heritage, 2012; Kimps, 2018; Labov, 1972). Tag questions are used in responsive actions to upgrade the speaker's claim to epistemic authority over their co‐participant with respect to the matter at hand (Heritage & Raymond, 2005) (e.g. Friend: ‘Your son has such dark brown eyes’. Mother: ‘He does, doesn't he’.). Tag questions are just one practice for displaying a stance about the rights and responsibilities of what participants know, or ought to know, and whether they have rights to describe it (Heritage & Raymond, 2005).

While tag questions (whether they have falling or rising intonation) are most often followed by a response (e.g. confirmation, disconfirmation or disagreement), they do not *require* a response (Hepburn & Potter, 2010; Kimps, 2018). A study that examined the relative frequencies of responses to tag questions found that 27% of tag questions were not responded to at all (Kimps, 2018). Tag questions are not typically used for requesting information or testing the recipient so, while they often project confirmation, they do not strictly require it for the conversation to progress (Hepburn & Potter, 2010). Due to this nonrequirement of a response, tag questions can be used in affiliative ways in contexts where a co‐participant may be unable or unwilling to contribute to the conversation at that time. For example, Hepburn and Potter (2010) found that tag questions often used by call operators on a child‐protection helpline occurred in sequences where the caller was crying (e.g. in response to a caller crying, the call‐taker responds ‘she's had a really difficult time hasn't she’.). The function of these tag questions was affiliative, validating the caller's epistemic authority and encouraging (but not requiring) participation from the caller at a point where their emotional expression may affect participation. Tag questions were found to be particularly useful for empathic receipts, where the anchor formulated the caller's emotional state, and ‘right‐thing descriptions’, where the anchor characterised the caller's course of action (Hepburn & Potter, 2010). Tag questions thus have an important characteristic that allows speakers to acknowledge the expertise of another party but without necessarily requiring that party to subsequently respond in relation to their expertise.

This study examines tag questions directed at child patients within paediatric palliative care consultations and how these tag questions orient to a child's epistemic authority over their own experiences. The findings are discussed in relation to the sociology of child agency and how this agency is acknowledged and displayed within and through social interaction.

## METHOD

This study was based on a corpus of 74 video recordings of paediatric palliative care consultations from three public hospitals in different Australian states. This corpus of recordings was collected as part of a research project aiming to understand how children are involved in conversations that profoundly affect them. Nine consultations were recorded as part of a pilot study in 2015. This pilot study established feasibility, and following the receipt of funding, the same method was used to record an additional 65 consultations in 2019–21. Consultations comprised 32 face‐to‐face outpatient consultations, 21 telehealth or telephone consultations (some of which were due to the COVID‐19 pandemic), 11 inpatient consultations and 10 home visits. Consultation length was an average of 43 minutes (SD = 20.1), with a total of 52 hours and 22 minutes hours of recorded data.

The study was approved by the Children's Health Services Queensland Human Research Ethics Committee (HREC/18/QRCH/86) and Queensland University of Technology Human Research Ethics Committee (1800000468), in addition to site‐specific governance approvals. The study adhered to the principles of the Australian National Health and Medical Research Council's National Statement on Ethical Conduct in Human Research.

### Participants

Participants for the study included 46 families and 49 clinicians working in paediatric palliative care (including doctors, nurses and various allied health professionals). The child patients ranged in age from 0–18 years old (M = 9 years, SD = 5.4) and 63% of the child patients were male (see Table 1). Diagnoses of the children included neurological conditions such as cerebral palsy, metabolic and other genetic disorders, cancer and other (often rare) conditions.

**TABLE 1:** Child patient demographics

Child	Sex	Age	Category	Diagnosis
F01	M	0.5	Neurology	Cerebral palsy
F02	M	17	Neurology	Duchenne muscular dystrophy
F03	M	17	Neurology	Duchenne muscular dystrophy
F04	M	7	Neurology	Schizencephaly
F05	M	1	Oncology	T cell lymphoblastic lymphoma
F06	F	9	Neurology	Cerebral palsy
F07	M	17	Neurology	Cerebral palsy
F08	M	10	Metabolic	Leukodystrophy
F09	F	6	Neurology	Cerebral palsy
F10	M	5	Neurology	Pontocerebellar hypoplasia
F11	F	14	Neurology	Cerebral palsy
F12	M	7	Metabolic	Mucopolysaccharidosis Type 3 (MPS III)
F13	M	15	Metabolic	Mucopolysaccharidosis Type 3 (MPS III)
F14	F	9	Neurology	Cerebral palsy
F15	M	3	Neurology	Epileptic encephalopathy
F17	M	3	Genetic	Phelan‐McDerind syndrome (22q13 microdeletion)
F18	M	3	Neurology	Epileptic encephalopathy
F19	F	13	Neurology	Cerebral palsy
F20	F	14	Genetic	CDKL5 deficiency disorder
F21	M	7	Metabolic	Lysosomal storage disorder
F22	M	5	Neurology	Epileptic encephalopathy
F23	M	9	Genetic	Lymphangiomatosis
F24	F	4	Neurology	Pontocerebellar hypoplasia
F25	M	4	Neurology	Genetic neurodevelopmental disorder
F26	F	3	Neurology	Cerebral palsy
F27	M	5	Cardiac	Congenital heart disease
F28	M	15	Multi‐system	Gastrointestinal dysmotility, autism, epilepsy
F29	M	8	Endocrine	Adrenal insufficiency
F30	F	12	Neurology	Cerebral palsy
F31	M	11	Neurology	Duchenne muscular dystrophy
F32	F	15	Neurology	Cerebral palsy
F33	F	16	Neurology	Undiagnosed neurodevelopmental disorder
F34	M	18	Neurology	Epileptic encephalopathy
F35	F	12	Genetic	CDKL5 deficiency disorder
F36	M	3	Metabolic	Leukodystrophy
F37	M	16	Neurology	Seizure disorder
F38	F	17	Neurology	Cerebral palsy
F39	M	2	Neurology	Merosin‐deficient muscular dystrophy
F40	F	8	Neurology	Batten's disease
F41	F	10	Neurology	Lissencephaly
F42	F	4	Oncology	Diffuse intrinsic pontine glioma
F43	M	0.25	Neurology	Myotubular myopathy
F44	M	3	Neurology	Genetic neurodevelopmental disorder
F45	F	15	Neurology	Juvenile Huntington's disease
F46	M	8	Neurology	Muscular dystrophy

### Procedure

Clinicians and families involved in the paediatric palliative care service were informed about the study by a member of the hospital staff. All participants provided informed written consent themselves or by their guardian (for child patients). Where possible, assent was additionally sought from children, although a child's age or condition often precluded this. Prior to the start of each recorded consultation, a clinician or research nurse set up two video cameras in the consultation room. No researchers were present during the video‐recorded consultations.

### Analysis

The focal phenomena were transcribed using the standard conversation analytic transcription conventions developed by Jefferson (2004) and Mondada (2018). The transcripts include details of pauses, overlapping talk, intonation and nonverbal communication found to be consequential for how participants manage social interactions. In the fragments presented, the following symbols were used for participants’ bodily‐behavioural actions:

+ = Parent/guardian action

∆ = Clinician action

* = Child action

The data were analysed using conversation analysis by authors KE, SE, LW and SD. This approach has longstanding application to the study of communication in clinical settings (Drew et al., 2001; Heritage & Maynard, 2006) including in paediatric palliative care (Ekberg et al., 2017, 2019, 2020). A conversation analytic approach uses observation to ensure analysis based on participants’ experiences and differs from other research approaches that begin with assumptions, intuitions or hypotheses (Sacks, 1984a). Analysis involves turn‐by‐turn examination of sequences of interaction to understand how specific conversational practices influence an ongoing interaction (Sidnell, 2013). For the analysis reported in this study, a collection was made of all tag questions directed to the child patient (*n* = 74 fragments). Each tag question was examined for its design and the action(s) it accomplished, which resulted in identification of the recurrent uses of and responses to tag questions. Pseudonyms are used in all transcribed data fragments that are reported in this study.

## FINDINGS

Across the corpus, tag questions directed at children were produced by adults (parents, guardians and clinicians) following a declarative claim about some matter that was ostensibly within a child's epistemic domain (i.e. something they are taken to have expertise about on the basis of knowledge or experience). As will be established across the analysis, the tag questions: (1) oriented to the child's epistemic authority over what was being said and (2) made a response from the child a possible but not necessary next action (i.e. because a response to a tag question is not a requirement). The tag questions were thus a practice that orients to a child's agency after someone else has ‘spoken for the child’ within an anchor statement.

Some tag questions were produced in relation to ‘AB‐events’, where the anchor related to something that both the speaker and the child have shared, common knowledge of, or experienced together (Heritage, 2012; Kimps, 2018; Labov, 1972). Other tag questions were produced in relation to ‘B‐events’, where the anchor related to something for which the child ostensibly is the ultimate epistemic authority (Heritage, 2012; Kimps, 2018; Labov, 1972). These AB‐event and B‐event tag questions are considered in turn in the following sections.

### *Tag questions produced in relation to shared AB*‐*events*

Fragments (1) and (2) provide examples of tag questions directed to a child following a telling by a parent to a clinician about an experience that was shared between the parent and child (i.e. an AB‐event). The child in Fragment (1) is three years old and has a diagnosis of epileptic encephalopathy. The child in Fragment (2) is seventeen years old and has a diagnosis of Duchenne muscular dystrophy. In each fragment, the anchor and tag question are highlighted in boldface.

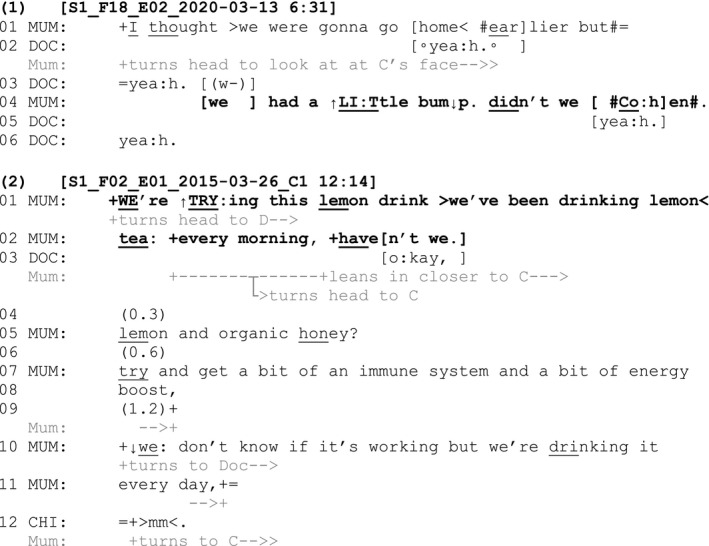

In Fragment (1), from an inpatient consultation, the mother is telling the palliative care doctor that they expected to go home from hospital earlier but they ‘had a little bump’ (line 4). Given it is both the child and the mother who are currently staying in the hospital and waiting to go home, this telling relates to a shared, common experience of the mother and child (i.e. an AB‐event). The mother uses ‘we’ to refer to her and the child across the turn and is gazing towards the child. She also adds a turn‐final tag directed to the child: ‘didn't we Cohen’ (line 4). The use of the child's name explicitly selects the child as a recipient, even though the telling was initially directed to the doctor. The design of the tag question thus orients to the child having shared epistemic accessibility to the claim that his mother has just made (Heritage & Raymond, 2005). At a moment where the child is not otherwise being incorporated into the conversation, the tag question orients to him as an active member of the interaction and having the opportunity to confirm or disconfirm his mother's claim. At the same time, the tag question does not necessitate a response from the child, so there is no burden for him to respond if he is not able or willing to do so. In this case, the child does not have capacity for verbal speech, and a verbal response would thus be unlikely.

In comparison with Fragment (1), which involved a child who does not have capacity for verbal speech, Fragment (2) involves a child who has such capacity. Similar to Fragment (1), the mother in Fragment (2) is telling the clinician that both she and her son (the child patient) have been drinking a lemon tea each morning. She uses ‘we’ across the turn to refer to her and the child and also adds the turn‐final tag ‘haven't we’ (line 2). Towards the end of her anchor turn, and prior to the tag, the mother shifts her gaze to the child and leans closer to him. Again here, the tag orients to the shared epistemic status of the child in relation to what is being told (they have both been drinking a lemon tea in the mornings) and provides him with the opportunity to confirm or disconfirm his mother's claim. Although the tag question is not responded to by the child, the mother pursues a response across lines 5–11, following which the child provides a verbal confirmation (‘mm’, line 12). These fragments demonstrate the use of tag questions in relation to AB‐events involving children who either have or do not have capacity for verbal speech.

### *Tag questions produced in relation to B*‐*events*

Tag questions were also commonly used following declarative claims that were made by adults about a child who ostensibly has the ultimate epistemic authority over anyone else in the room about the matter being discussed (i.e. a B‐event). These tag questions sometimes directly addressed the child after they have been earlier referred to in the third person (e.g. using pronouns such as ‘he’ and ‘she’). This can be seen in Fragments (3) and (4) below. The child in these fragments is seven years old and has lysosomal storage disorder. The reference to ‘NDIS’ in Fragment (4) relates to Australia's National Disability Insurance Scheme.

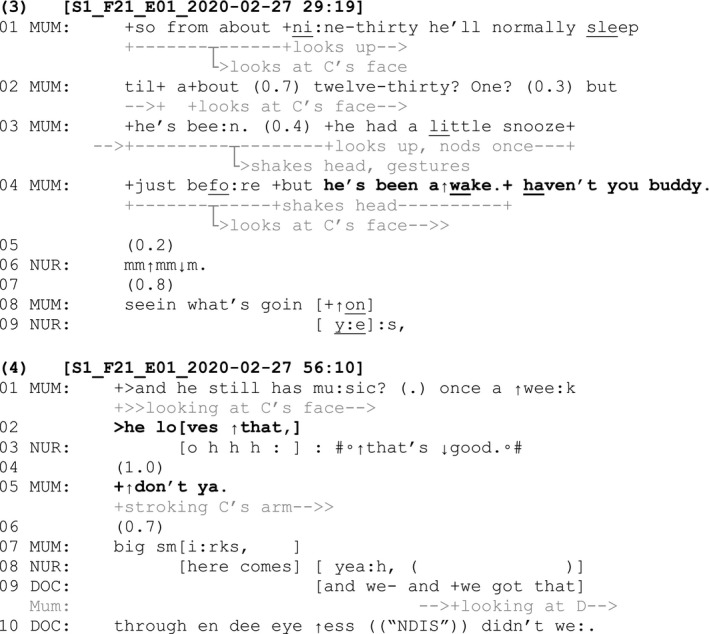

In each of these fragments, a declarative claim has been made about the child, who is referred to in the third person (‘he's been awake’, line 4, Fragment 3; and ‘he loves that’, line 2, Fragment 4). For each claim, it is the child who ostensibly is the ultimate epistemic authority. In each case, the child's epistemic authority is acknowledged through the addition of a tag directed to the child (‘haven't you buddy’, line 4, Fragment 3; and ‘don't ya.’, line 5, Fragment 4). The tag questions position the child as an active member of the interaction and provide an opportunity for the child to contribute to the conversation if they are willing or able to do so. In Fragment 4, the mother's tag is added as an increment, an utterance added to a turn that is already possibly complete (Schegloff, 2016). This incremental addition of the mother's tag question occurs after the nurse has already responded to the assessment ‘he loves that’ (lines 2–3), and there has been a one second gap in the conversation (line 4). Even though the sequence has progressed without ostensible trouble, the subsequent use of a tag orients to the child having greater epistemic authority over the claim that has been made about his love of music lessons. Following another 0.7‐second gap (line 6), the mother adds evidence for her secondhand knowledge on this matter: the fact that he has ‘big smirks’ on his face during music lessons (line 7). This addition of evidence highlights that the mother's knowledge of her claim is based on observing the child's behaviour and is thus secondary knowledge to the child's knowledge of his own feelings during music lessons.

On other occasions, tag questions related to B‐events were produced following a declarative directed to the child (‘you’) as seen in Fragments (5) and (6) below. The child in Fragment (5) is seven years old and has a diagnosis of schizencephaly.

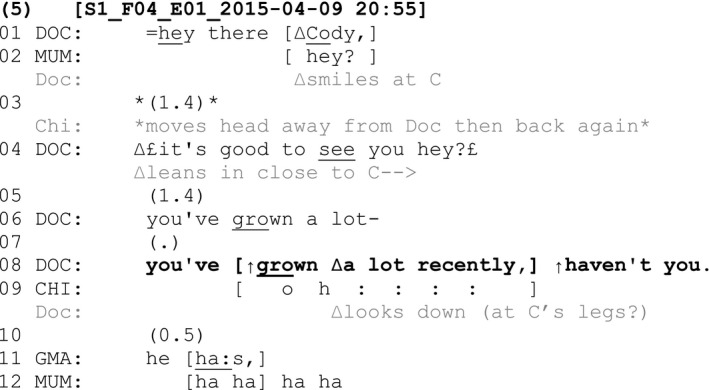

In Fragment (5), the child has just woken from sleeping during the consultation and the clinician greets the child. Following this greeting, the clinician makes a declarative claim directed to the child: ‘You've grown a lot recently’ (line 8). He looks at the child across the turn. This assessment is within a context where the child is the primary epistemic authority. It would not be new information for the child—while the clinician is only just seeing the child today, and the child would likely already know he has grown recently. Given the putative rule that people should not be told things that they are supposed to already know (Sacks, 1995; Schegloff, 2007), orienting to the child's epistemic authority is possibly relevant. This is accomplished through a tag question at the end of the clinician's turn: ‘haven't you’ (line 8). The tag question directed to the child provides the child with an opportunity to respond—whether verbally, vocally, or body‐behaviourally—without requiring such responses from him. The child is thus treated as an active, knowledgeable conversational partner. There is a 0.5‐second pause before the child's grandmother responds with a confirmation ‘he has’ (line 11). This is a nonconforming confirmation insofar as it does not conform to the constraints set by the preceding tag question (Heritage & Raymond, 2005), which selects the patient as the recipient. The grandmother also asserts her epistemic stance over the clinician's assessment as someone who sees the child every day and would also know about his growth (Raymond & Heritage, 2006).

The next fragment provides an example of a child disconfirming an assessment about a B‐event that is followed by a tag question, thus asserting his epistemic authority in response. The child in this fragment is seventeen years old and has a diagnosis of Duchenne muscular dystrophy.

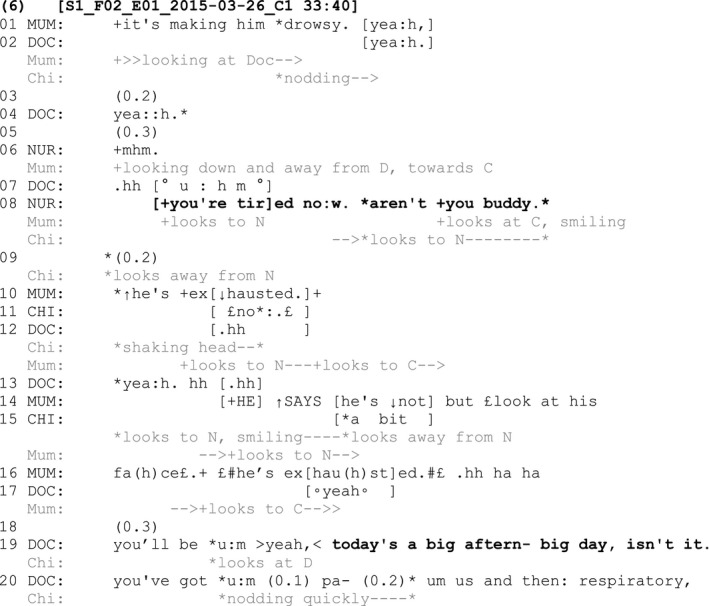

In this fragment, the nurse directs an assessment about the child to the child (‘You're tired now’, line 8) and follows this with a tag (‘aren't you buddy’, line 8). The child and his mother both respond to the nurse at the same time, but with different responses. The mother confirms ‘he's exhausted’ (line 10), claiming epistemic authority to respond on behalf of the child. The child, however, shakes his head in overlap and follows up with a verbal response using smile voice ‘no’ (line 11). The child here thus displays a right to disconfirm the nurse's assessment of him, which is a way of displaying his epistemic authority over this matter. He subsequently qualifies his previous response by adding ‘a bit’ (line 15). The mother manages the child's conflicting response by implying the child is not telling the truth and adding evidence for her claim that he is exhausted: ‘He says he's not but look at his face he's exhausted’ (lines 14 and 16). Rather than aligning with either party, the doctor makes a different assessment that ‘today's a big…day’ (line 19) and adds a tag question ‘isn't it’ (line 19) before listing the different hospital services that the child is visiting for appointments that day. The child nods in response to the doctor's tag question, again displaying recognition of his epistemic authority in relation to this new claim and actively contributing to the conversation to provide confirmation in response.

### Deviant cases

The following two fragments show instances where a tag question is not initially used following a declarative claim that relates to knowledge or experience about which the child ostensibly has ultimate epistemic authority. In other words, while the child is present in the interaction, an adult speaker's turn does not orient to the child's epistemic authority over the anchor statement. In both these instances, the absence of a tag question (and therein an orientation to the child's epistemic authority) is managed in the interaction in a way that a tag question is ultimately then produced in a subsequent turn. Fragment (7), involving a seventeen‐year‐old child with Duchenne muscular dystrophy, provides the first example of this practice:

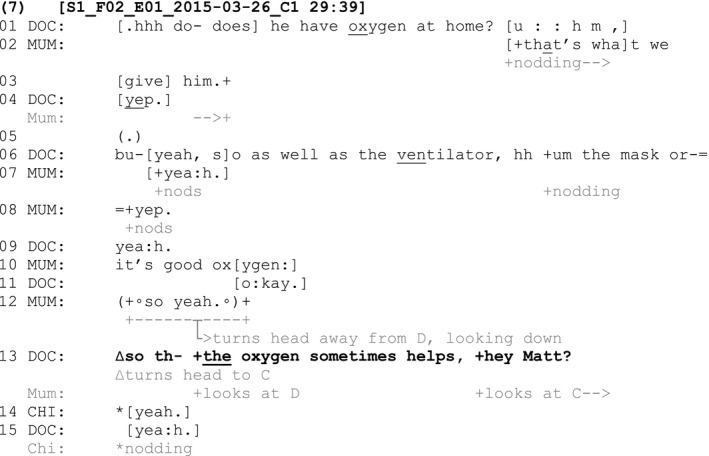

At line 1, the doctor asks the mother a question about whether the child has oxygen at home. The mother confirms that he does and, following a check from the doctor and further confirmation (lines 6–8), the mother provides an assessment that ‘it's good oxygen’ (line 10). This assessment is something that the child has ultimate epistemic authority over as it is he who receives the oxygen to relieve pain and breathing difficulties. The child's epistemic authority in relation to this B‐event is not acknowledged. His mother does not gaze at him or add a tag question to her turn, as seen in the examples analysed so far. The doctor first provides an acknowledgement at line 11 and then turns to the child and provides a gist formulation (Antaki, 2008), which rephrases the mother's assessment (‘so the oxygen sometimes helps’, line 13), followed by a tag question (‘hey Matt?’, line 13). As well as gazing at the child across the turn, the doctor uses the child's name, thus explicitly displaying the child as the selected recipient of his turn. By adding this gist formulation and a tag question, the doctor manages the mother's lack of orientation to the child's epistemic authority over her claim. The doctor does this orientation by reproducing the assessment with a tag directed to the child. In doing so, the doctor orients to the child's ongoing status as an active participant in the conversation, providing an opportunity for the child to confirm or disconfirm the claim that has been made about him. The child provides confirmation verbally (‘yeah’, line 14) and nonverbally (nodding) to the doctor in response to the doctor's tag question. This fragment provides an example where an adult (in this case, the parent) makes a claim about a B‐event in the presence of the child but does not use a tag question to orient to the child's epistemic authority over the claim. The lack of a tag question is then managed by the other adult in the interaction (in this case, the clinician) who reformulates the turn and adds the tag question directed to the child.

Another deviant case example can be seen in Fragment (8) with a nine‐year‐old child with cerebral palsy, who communicates in the below fragment using a voice output communication aid:

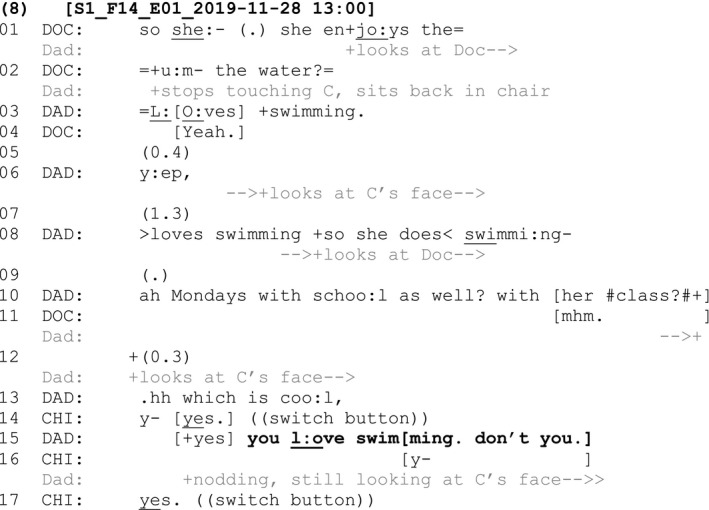

At lines 1–2, the doctor asks the child's father if she enjoys the water. The father replies that the child ‘loves swimming’ (line 3), expands his response by adding that she does swimming lessons with her school class on Mondays and then adds the increment ‘which is cool’ (line 13) to his otherwise possibly complete turn. The father gazes at the child periodically across his extended turn but does not explicitly orient to the child's epistemic authority the claims about swimming (lines 8, 10 and 13). At line 13, the child initiates a turn by pressing the ‘yes’ switch button on her voice output communication aid (VOCA). The child thus displays her right to confirm the claims made by her father, even though she was not explicitly invited to within the father's prior turns. This initiation displays that she recognises her own agency in relation to the topic of conversation. In response to the child's confirmation, the father partially repeats his claim about swimming, but this time adds a tag ‘don't you?’ (line 15). The child repeats her confirmation ‘yes’ on her VOCA. So, in a similar way to Fragment 7, an adult's turn includes a claim about a B‐event without orienting to the child's epistemic authority over that claim. In this example, it is the child herself who draws attention to this lack of orientation to her epistemic authority in a subsequent turn. The child's self‐initiated confirmation occasions a repeat of the claim from the father, this time acknowledging his downgraded epistemic authority and orienting to her agency by adding a tag question inviting (re)confirmation from her.

These two deviant cases highlight how both adults and children in the conversations orient to a child having ultimate epistemic authority over claims being made in a consultation. In both cases, this orientation is made through the repetition of a previous claim, followed by the inclusion of a tag question that acknowledges the child's ultimate epistemic authority in relation to that claim.

## DISCUSSION

This study focussed on an examination of clinicians’ and parents’ tag questions directed at child patients within paediatric palliative care consultations. The findings demonstrated how the adults in the consultation used tag questions when making an anchor claim that was ostensibly within the child's epistemic domain. More specifically, tag questions were used when claims were made that were related to either an AB‐event, of which both the speaker and the child had shared or common knowledge, or a B‐event, where the child had ultimate epistemic authority. The use of tag questions is a means by which an adult speaker can acknowledge a child's epistemic authority over what was being said. These tag questions also invited participation from the child, whether verbally, vocally or body‐behaviourally, without making any absence of such participation accountable (i.e. because a response to a tag question is not a necessary requirement). Tag questions were thus a way to manage ‘speaking for a child’ when that child was present but not necessarily able or willing to extensively contribute to the conversation.

Tag questions like those considered in this study can be seen to occupy a middle ground for involving children in interaction. They occasion an opportunity where a child might confirm or disconfirm an anchor statement, thereby orienting to the child as an active member of the interaction. Indeed, the children in this study did sometimes respond to the tag questions with confirmations or even disconfirmations, demonstrating their ability and willingness to respond. At the same time, because a response was not an interactional necessity for the conversation to proceed, the tag questions did not place a burden of responding on the child in a way that a more open, information‐soliciting question might do. The use of tag questions in this environment builds on prior research by Clemente (2009) and Clemente et al. (2012), who showed that practices such as closed‐ended questioning place fewer demands on the child, which may help to reduce the risk of no response, or a parent responding instead of the child.

Instances when children did respond to tag questions (or provided confirmations in an environment where a tag question was not initially used, as in Fragment 8) highlighted that children displayed their primary access to and right to comment on their own subjective experience. This finding supports previous conversation analytic research that showed direct evidence of children's competence as informants about their own experience within health‐care settings (Clemente et al., 2008; Jenkins, 2015; Jenkins et al., 2020). Given that previous research has shown that children typically only speak in 5% of the conversation within health‐care consultations (Cahill, 2010; Cahill & Papageorgiou, 2007), these findings provide a nuanced understanding of an existing practice that highlights a way that children can be involved in a conversation.

More broadly, the findings support the new sociology of childhood that treats children as active in the construction and determination of their own social lives rather than passive subjects of social structures and processes (Moran‐Ellis, 2010; Prout & James, 2015). Examining interaction within these health‐care appointments showed how adult participants oriented to children's agency when describing shared experiences or secondhand, observed experiences of the child. It also showed how children were aware of and competently employed their own agency in responding to adults’ descriptions of these experiences. These findings thus build on prior conversation analytic research in health‐care and other institutional settings that provide direct evidence for how children's agency is co‐constructed through unfolding sequences of talk. This encompasses instances when children do and do not speak, and at points where they may or may not have been selected to speak (e.g. Church & Bateman, 2019; Clemente, 2009, 2015; Clemente et al., 2008, 2012; Danby, 2002; Houen et al., 2016; Hutchby, 2002; Jenkins et al., 2020).

### Practice implications

The use of tag questions directed at the child might be particularly useful in the context of paediatric palliative care with children with complex conditions who may not always have the ability or willingness to contribute extensively to a verbal conversation. The data in this study showed that at least some of these children display understandings that particular experiences are primarily within their epistemic domain and when they have rights over these interactional descriptions of these experiences. The findings lend support for child patients in paediatric palliative care to be encouraged to contribute to consultations wherever possible, including where this requires technology such as a voice output communication aid (VOCA) to do so. Tag questions are one way that clinicians and parents/guardians can acknowledge and encourage contributions from child patients without making any absence of such participation accountable.

### Strengths and limitations

This is the first study to directly analyze how clinicians and parents/guardians direct talk towards a child patient in real‐life, video‐recorded paediatric palliative care consultations. It adds to the small, and growing, body of research examining naturally‐occurring communication within paediatric palliative care (Ekberg et al., 2017, 2019). A limitation of the study was that data were collected from three public hospitals in Australia. Future research might seek to collect data at additional sites and locations.

The findings of the current study highlight fruitful areas for future analysis, which could be pursued through further analysis of the data collected for this study and through research conducted using other corpora of data. This future research should consider other communication practices that acknowledge children's agency and provide opportunities for child patients to be participants in the interaction during health‐care consultations, in a manner of their choosing. In particular, more research is needed examining communication practices that engage children with complex conditions within settings such as paediatric palliative care.

## AUTHOR CONTRIBUTION

**Katie Ekberg:** Conceptualization (lead); Data curation (equal); Formal analysis (lead); Investigation (equal); Methodology (equal); Project administration (lead); Supervision (supporting); Writing – original draft (lead); Writing – review & editing (lead). **Stuart Ekberg:** Conceptualization (supporting); Data curation (supporting); Formal analysis (supporting); Funding acquisition (supporting); Investigation (supporting); Methodology (supporting); Supervision (supporting); Writing – original draft (supporting); Writing – review & editing (supporting). **Lara Weinglass:** Data curation (supporting); Formal analysis (supporting); Investigation (supporting); Methodology (supporting); Writing – review & editing (supporting). **Anthony Herbert:** Conceptualization (supporting); Data curation (supporting); Funding acquisition (supporting); Resources (supporting); Writing – review & editing (supporting). **Johanna Rendle‐Short:** Conceptualization (supporting); Formal analysis (supporting); Funding acquisition (supporting); Methodology (supporting); Writing – review & editing (supporting). **Myra Bluebond‐Langner:** Conceptualization (supporting); Funding acquisition (supporting); Investigation (supporting); Writing – review & editing (supporting). **Patricia Yates:** Conceptualization (supporting); Funding acquisition (supporting); Writing – review & editing (supporting). **Natalie Bradford:** Conceptualization (supporting); Funding acquisition (supporting); Writing – review & editing (supporting). **Susan Jill Danby:** Conceptualization (supporting); Data curation (supporting); Formal analysis (supporting); Funding acquisition (lead); Investigation (supporting); Methodology (supporting); Project administration (lead); Supervision (lead); Writing – review & editing (supporting).

## Data Availability

Authors elect to not share data due to sensitivity of data and ethical requirements.
